# Severe muscle pain and stiffness due to dexmethylphenidate

**DOI:** 10.1002/ccr3.2628

**Published:** 2020-01-25

**Authors:** Jacob J. Kon, Alexander A. Kon

**Affiliations:** ^1^ Department of Pediatrics University of California San Diego School of Medicine San Diego CA USA

**Keywords:** attention deficit hyperactivity disorder, central nervous system stimulants, myalgia

## Abstract

Dexmethylphenidate, and potentially other methylphenidates used in the treatment of attention deficit hyperactivity disorder (ADHD), may cause severe muscle pain and stiffness. Medication side effects should be considered as the possible cause if a patient with ADHD develops severe symptoms.

## Graphical abstract

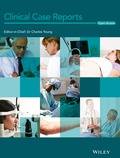

## 1 INTRODUCTION

Attention deficit hyperactivity disorder (ADHD) is a significant healthcare problem effecting approximately 10% of American youth.^1^, ^2^, ^3^ With growing rates of ADHD, diagnosis and treatment is becoming common in general pediatric and family practice settings. As general pediatricians and family physicians become increasingly responsible for prescribing stimulant medications, it is imperative that they are cognizant of the potential side effects, including uncommon but significant side effects, to ensure excellent care of their patients.

A well‐described side effect of stimulant medications is muscle twitching. Further, stimulant medications can lead to the serotonin syndrome, which may include severe muscle twitching, rigidity, and loss of muscle coordination in the face of agitation, confusion, tachycardia, and other serotonin‐related symptoms. The amphetamine stimulant lisdexamfetamine has been reported to cause muscle pain, cramping, and stiffness without other serotonin‐related symptoms.^4^, ^5^ While these side effects have been most widely reported with lisdexamfetamine, there are data suggesting that the combination medication dextroamphetamine‐amphetamine (also an amphetamine) can cause similar symptoms.^6^ Muscle pain, cramping, and stiffness without other serotonin‐related symptoms have not been reported for non‐amphetamine stimulant medications used in the treatment of ADHD, including the methylphenidate class of medications. We report a case of severe muscle pain and stiffness caused by dexmethylphenidate, which is previously unreported.

## 2 CASE PRESENTATION

Kyle (pseudonym) was a 12‐year‐old previously healthy boy diagnosed with ADHD (predominantly inattentive subtype) who was noted to have slightly increased muscle tone and slightly decreased range of motion of the limb girdles at baseline; however, he had never required physical therapy or workup. Kyle was prescribed dexmethylphenidate extended release 5 mg daily to assist with school performance. Over several months, Kyle's dose was gradually increased based on his suboptimal ability to concentrate at school. With increasing doses, Kyle's ADHD control improved; however, he continued to have insufficient control in spite of dose increase up to 30 mg daily. Over the same period of time, Kyle began to complain of increasing muscle pain and stiffness predominantly of the thighs bilaterally; however, the etiology of his symptoms was unclear. Over time, the symptoms became so severe that he was unable to participate in physical education at school or in afterschool sports, and eventually, he experienced significant pain walking even short distances. Kyle also complained of severe stiffness of his muscles, which seemed worse in the mornings, and parents reported finding him crying in bed at night due to severe thigh pain. Kyle was assessed for possible serotonin syndrome; however, he showed no clonus, agitation, diaphoresis, hyperreflexia, or fever; therefore, serotonin syndrome was ruled out.^7^ Because such symptoms in the absence of serotonin syndrome had not been reported with dexmethylphenidate, the medication was not felt to be the cause of the symptoms.

Due to the severity of symptoms, Kyle was referred to pediatric rheumatology, metabolic clinic, and child neurology. All laboratory and radiographic testing was within normal limits: ANA negative; LDH total 172 U/L (LDH 1 21%; LDH 2 31%; LDH 3 24%; LDH 4 12%; LDH 5 12%); CK 92 units/L; albumin 4.2 g/dL; total bilirubin 0.3 mg/dL; direct bilirubin < 0.1 mg/dL; Alk Phos 286 units/L; AST 26 units/L; ALT 28 units/L; total protein 7.5 g/dL; aldolase 3.9 U/L; C‐reactive protein <3.0 mg/L; sedimentation Rate 2 mm/h; hip and pelvis X‐rays within normal limits. Subspecialty referral yielded no diagnosis. Kyle was seen by physical therapy due to persistent goniometer assessment demonstrating decreased range of motion in the hips bilaterally and was prescribed stretching and strength training; however, this did not improve his symptoms. Eventually, it was noted that the symptoms had become intolerable approximately one week after the dexmethylphenidate had been increased to 30 mg daily; therefore, a trial‐off medication was attempted.

Kyle's symptoms improved dramatically within 5 days of stopping the dexmethylphenidate. After two weeks off of the medication, Kyle was again able to participate in physical education and sports. Although Kyle was not back to baseline, the dexmethylphenidate was restarted at 5mg daily after a month off medication due to difficulty concentrating. With the lower dose, the symptoms remained stable. Due to ongoing stiffness and pain, although significantly better than on the higher dose of medication, Kyle chose to discontinue stimulant medications. The muscle pain resolved several weeks after medication discontinuation. Muscle stiffness, treated with physical therapy, slowly improved, and patient was at baseline approximately 6 months after stopping medication.

## 3 DISCUSSION

Muscle pain and stiffness have been reported in some patients with untreated ADHD,^8^ and stimulant medications have been reported to improve muscle tension in some of these patients.^9^ Conversely, lisdexamfetamine is well‐known to cause muscle pain and stiffness in some patients, and there are data suggesting that the combination medication dextroamphetamine‐amphetamine may do so as well.^4^, ^5^, ^6^ Both lisdexamfetamine and dextroamphetamine‐amphetamine are amphetamine stimulant medications; therefore, similar activity of these medication may be expected. Other non‐amphetamine stimulant medications (such as the methylphenidates), however, have not previously been implicated in causing significant muscle pain and stiffness without other serotonin‐related symptoms. An extensive literature review produced only a single case in which a child developed pain on methylphenidate, and that child presented with a constellation of diplopia, abdominal pain, and leg pain.^10^

Amphetamines primarily increase the release of dopamine and norepinephrine, and to a lesser extent decrease the rate of reuptake of these neurotransmitters.^11^, ^12^, ^13^ In contrast, the methylphenidate medications primarily function as neurotransmitter reuptake inhibitors with only a minor increase in their release.^3^, ^12^ It is possible that this difference in mode of action explains the higher rate of symptoms of muscle pain and stiffness in the amphetamine stimulant medication compared to the methylphenidate medications. Dopamine is well‐known to be a key neurotransmitter; however, there is evidence that dopamine also directly modulates muscle tone.^14^ Based on the direct effect dopamine may have on skeletal muscle, the muscle pain and stiffness reported previously with amphetamine stimulants, and here with a methylphenidate medication, may be due to over‐stimulation of skeletal muscle fibers by excessive dopamine.

While increased direct effect of dopamine on skeletal muscles may explain increased muscle tension, twitching, stiffness, and potentially pain, our patient experienced severe pain which woke him at night. It seems unlikely that direct dopamine effects would be solely responsible for this level of pain. We postulated that the severe muscle pain experienced by our patient may have been due to local rhabdomyolysis. Amphetamines are known to cause rhabdomyolysis even with a single dose.^15^, ^16^, ^17^, ^18^ In general, however, rhabdomyolysis associated with amphetamine use is caused by vigorous muscular exercise, hyperthermia, cellular hypermetabolism, reduced muscle perfusion, coagulopathy, and systemic hypotension.^19^ Amphetamines can, however, directly activate skeletal muscle thermogenic protein, potentially causing local thermogenesis in large skeletal muscle groups.^19^ This occurs through a mitochondrial uncoupling protein, which disassociates the mitochondrial proton gradient from ATP synthesis, thereby releasing free energy as heat into the skeletal muscle cell.^19^ Further, amphetamines can have a direct vasoconstriction effect^19^ which may decrease the ability of affected muscle fibers to clear toxins and dissipate heat. As such, it may be possible for amphetamine stimulant medications at high doses to cause sufficient local, intracellular hyperthermia to cause mild, localized rhabdomyolysis without generalized hyperthermia and widespread rhabdomyolysis, which may explain localized muscle pain in patients taking amphetamine stimulant medications. Research has not been performed to assess whether methylphenidate stimulants have similar effects on uncoupling proteins and local vasoconstriction. Research in this area would be helpful to better understand the symptomatology of our patient.

We also note that our patient had a long‐standing history of poor flexibility. Further, parents report a significant family history of poor flexibility in one parent's lineage. It is therefore possible that the family history points to a genetic predisposition for this syndrome. Parents are Caucasian of Ashkenazi Jewish descent; no HLA‐typing was performed.

## 4 CONCLUSION

General pediatricians and family physicians are increasingly prescribing stimulant medications for children with ADHD. Muscle pain, cramping, and stiffness have been previously reported with amphetamine stimulant medications; however, methylphenidate stimulants have been considered less likely to cause these symptoms. We report an adolescent male with severe muscle pain and stiffness due to methylphenidate therapy. Primary care providers should be aware of this rare but significant side effect and should consider a trial‐off medication for patients who present with these symptoms prior to referral and extended workup.

## CONFLICTS OF INTEREST

The authors have no conflicts of interest relevant to this article to disclose.

## AUTHOR CONTRIBUTIONS

JK: performed the background research, wrote the first draft of the manuscript, and reviewed and approved the final draft. AK: conceived of the project, edited the manuscript, and reviewed and approved the final draft.
